# Combined Effects of Vegetable Oil-, Micronutrient-, and Activated Flavonoid-Based Biostimulants on Photosynthesis, Nematode Suppression, and Fruit Quality of Cucumber (*Cucumis sativus* L.)

**DOI:** 10.3390/plants15020274

**Published:** 2026-01-16

**Authors:** Georgia Ouzounidou, Niki-Sophia Antaraki, Antonios Anagnostou, George Daskas, Ioannis-Dimosthenis Adamakis

**Affiliations:** 1 Institute of Technology of Agricultural Products, ELGO-DIMITRA, 14123 Lycovrissi, Greece; antony.anagnostou@gmail.com; 2 Section of Botany, Department of Biology, National and Kapodistrian University of Athens, 15784 Athens, Greece; nikintrk1504@gmail.com (N.-S.A.);; 3 Geogreen Marathon, 235-237 Marathonos Street, 19007 Marathonas, Greece; geogreenmarathon@gmail.com

**Keywords:** *Cucumis sativus* L., biostimulants, photosynthesis, electron transport, oxygen-evolving complex (OEC), nematodes, galls, giant cells, syncytia

## Abstract

The agricultural industry faces increasing environmental degradation due to the intensive use of conventional chemical fertilizers, leading to water pollution and alterations in soil composition. In addition, root-knot and cyst nematodes are major constraints to cucumber production, causing severe root damage and yield losses worldwide, underscoring the need for sustainable alternatives to conventional fertilization and pest management. Under greenhouse conditions, a four-month cultivation trial evaluated vegetable oil-, micronutrient-, and activated flavonoid-based biostimulants, applying Key Eco Oil^®^ (Miami, USA) via soil drench (every 15 days) combined with foliar sprays of CropBioLife^®^ (Victoria, Australia) and KeyPlex 120^®^ (Miami, USA) (every 7 days). Results showed reduced parasitic nematodes by 66% in soil and decreased gall formation by 41% in roots. Chlorophyll fluorescence and infrared gas analysis revealed higher oxygen-evolving complex efficiency (38%), increased PSII electron transport, improved the fluorescence decrease ratio, also known as the vitality index (Rfd), and higher CO_2_ assimilation compared to conventional treatments. Processed cucumbers showed higher sugar and nearly double ascorbic acid content, with improved flesh consistency and color. Therefore, the application of these bioactive products significantly reduced nematode infestation while enhancing plant growth and physiological performance, underscoring their potential as sustainable tools for crop cultivation and protection. These results provide evidence that sustainable bioactive biostimulants improve plant resilience, productivity, and nutritional quality, offering also an environmentally sound approach to pest management.

## 1. Introduction

Modern agriculture is confronted with critical challenges that must be addressed to ensure global food security. Current estimates indicate that the world’s population exceeds 7 billion, and projections suggest it will reach approximately 9.7 billion by 2050 [1]. This demographic expansion is expected to increase food demand by 25–70% relative to present production levels. Concurrently, agriculture is both impacted by and contributes to climate change. Extreme weather events, including heatwaves and cold spells, are increasingly associated with global warming [2,3], while agricultural activities themselves are significant sources of greenhouse gas emissions. Addressing these dual pressures is essential to sustain and expand food production.

Approximately 40% of arable land worldwide is affected by reduced fertility, and further land conversion to meet rising demand threatens plant and animal biodiversity. This scenario underscores the urgent need to enhance crop yields while minimizing environmental impacts. One promising approach involves the use of plant biostimulants, which have emerged as a strategy to promote sustainable agricultural practices. The European regulatory framework defines biostimulants as products evaluated not by their nutrient content but by their functional effects, which include improved nutrient uptake and use efficiency, enhanced tolerance to abiotic stress, and superior crop quality [4,5]. In contrast, regulatory authorities in the United States have yet to establish a formal definition, although a pending proposal closely aligns with the European model [6].

Biostimulants encompass a diverse range of active substances that promote plant health and growth. These include microorganisms (e.g., plant growth-promoting rhizobacteria and mycorrhizal fungi), plant- and algal-derived extracts, protein hydrolysates, humic and fulvic acids, polysaccharides, micronutrients, vegetable oils, and activated flavonoids [7,8]. Depending on their formulation and intended use, biostimulants may be applied to soil, the rhizosphere, or directly to foliage. Their efficacy derives from the activation and regulation of key metabolic and physiological processes, such as stimulation of root system architecture, enhancement of photosynthetic capacity, improved nutrient uptake and assimilation efficiency, modulation of phytohormone balance, and induction of plant defense responses against biotic and abiotic stressors [9,10].

Recent studies have shown that biostimulants can positively influence photosystem II (PSII) efficiency, electron transport rates, chlorophyll content, and carbon assimilation, thereby improving plant resilience under stress conditions such as nutrient deficiency, salinity, drought, and pathogen pressure [11,12]. Flavonoid- and micronutrient-based formulations have been reported to enhance antioxidant activity and protect the photosynthetic apparatus from oxidative damage, contributing to sustained photosynthetic performance and biomass accumulation [13].

Collectively, these effects contribute to improved plant performance, increased agricultural productivity, and greater sustainability by reducing reliance on synthetic fertilizers and chemical plant protection products. The capacity of plants to achieve and maintain high photosynthetic efficiency is strongly influenced by both biotic and abiotic stress factors. Ensuring optimal photosynthesis performance in food crops under adverse environmental conditions remains a major challenge for crop breeders and plant scientists, especially in the context of climate change and the increasing global demand for food [14,15,16]. In this regard, biostimulants represent a promising complementary strategy for enhancing crop productivity while supporting environmentally sustainable agricultural systems.

Cucumber (*Cucumis sativus* L.), a member of the *Cucurbitaceae* family, is recognized as a high-value horticultural crop and is widely cultivated under protected environments such as greenhouses [17]. Cultivation in greenhouses is common practice to mitigate adverse environmental conditions, particularly wind, which can cause fruit abrasion against thorny leaves and stems, leading to blemishes and scars that reduce marketability. Cucumber is a widely consumed salad vegetable, valued both for fresh consumption and for preservation. From a nutritional perspective, the edible portion of cucumber contains approximately 0.4% protein, 2.5% carbohydrates, and 0.1% fat, along with 7.0 mg of vitamin C, 25 mg of phosphorus, 10 mg of calcium, and 1.5 mg of iron per 100 g of fresh fruit. In addition, cucumbers are a source of vitamins C, E, and B-complex, including folic and pantothenic acids [17].

Root-parasitic nematodes represent one of the most persistent and damaging constraints in intensive horticultural systems, especially in crops such as *Cucumis sativus* L. [18]. Among the most economically important groups are the root-knot nematodes (*Meloidogyne* spp.) and cyst nematodes (*Heterodera* spp.), both of which establish highly specialized feeding structures inside host roots [19,20]. These sedentary endoparasites induce profound anatomical rearrangements: Meloidogyne species stimulate the formation of giant cells and prominent galls, whereas *Heterodera* spp. generate syncytia—multinucleated, highly metabolically active feeding domains [20]. These structures divert assimilates and disrupt water and nutrient transport, leading to chlorosis, stunting, reduced fruit quality, and ultimately yield loss [18]. Their persistence in soil, high reproductive rates, and ability to withstand environmental fluctuations render traditional chemical nematicides increasingly ineffective [21]. Moreover, the ecological cost of nematicides—loss of beneficial microflora, environmental contamination, and selection for resistant nematode populations—has intensified the demand for sustainable biological tools [22]. Biostimulants derived from flavonoids, plant oils, and humic complexes are emerging as promising alternatives due to their ability to enhance plant physiology, strengthen defense responses, and improve soil ecological balance, ultimately offering a more resilient approach to nematode management [23].

Despite growing evidence supporting the beneficial effects of individual biostimulant categories—such as flavonoids, botanical oils, humic substances, or micronutrients—most existing studies have focused on single-component applications or evaluated plant growth responses without addressing their combined regulatory effects [5,8]. Consequently, a significant knowledge gap remains regarding how integrated biostimulant formulations influence cucumber photosynthetic performance, nematode resistance, fruit quality, and soil biological dynamics under greenhouse conditions. Understanding whether these effects are driven primarily by plant-mediated resistance or also involve broader modulation of the soil ecosystem is essential for advancing sustainable crop management strategies.

In response to escalating environmental challenges associated with climate change, the adoption of sustainable agricultural practices has become increasingly vital, particularly within organic fruit and vegetable production systems [24]. Taking these considerations into account, the present study was designed to evaluate the growth performance of cucumber plants cultivated under a defined biostimulant protocol in comparison with conventional practices. Beyond plant growth, emphasis was placed on key physiological performance. The impact of combined biostimulant applications (flavonoids + botanical oils + humic acids + micronutrients) on cucumber photosynthetic efficiency, chlorophyll fluorescence, and assimilation rates under greenhouse conditions was determined. In addition, the evaluation of cucumber fruit nutritional composition under biostimulant treatments compared to conventional practices, while nematode resistance and root health by assessing the effectiveness of the biostimulant protocol in reducing root-parasitic nematode damage, improving root anatomical integrity, and mitigating pathological symptoms were studied.

To achieve a more comprehensive understanding of biostimulant effects, the study also incorporated detailed soil extraction analyses and root anatomical assessments. These evaluations revealed clear differences between the control and biostimulant-treated plots in both the density and composition of nematode communities, as well as in their corresponding impacts on root morphology. Although total nematode abundance remained similar between treatments, biostimulant application—particularly with CropBioLife^®^, KeyPlex 120^®,^ and Key Eco Oil^®^—resulted in substantial reductions in plant-parasitic nematodes, alongside improved root structure and fewer pathological symptoms. Moreover, shifts in non-parasitic nematode groups suggested a more balanced soil ecological profile under biostimulant treatment. It is worth noting that these observations highlight the broader agronomic significance of biostimulants, demonstrating their potential not only to enhance plant physiological performance and fruit quality but also to foster a healthier soil ecosystem that supports more resilient and functional root systems. Overall, this study uniquely integrates photosynthetic performance, nematode suppression, and fruit quality under biostimulant treatment—an intersection not previously addressed.

## 2. Results

### 2.1. Soil Analysis

Post-experiment soil analyses indicated that key physicochemical parameters, including pH, electrical conductivity, organic matter content, total nitrogen, and microbial quotient (qMIC), remained stable throughout the cultivation period. No statistically significant differences were observed between control and biostimulant-treated soils at the end of the experiment (*p* > 0.05). Nevertheless, biostimulant-treated plots exhibited slight, non-significant increases in organic matter, total nitrogen, and qMIC, suggesting a tendency toward enhanced microbial activity and improved soil biological balance without inducing adverse chemical alterations (Table 1).

### 2.2. Photosynthesis

**Figure 1:**
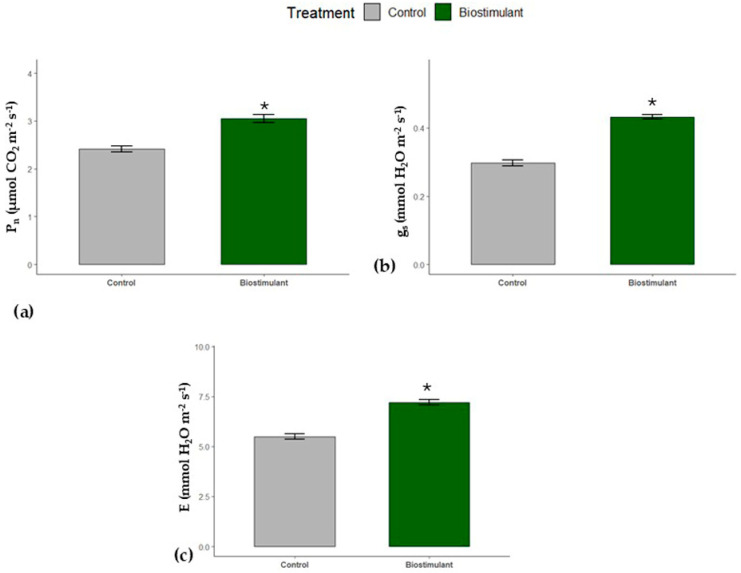
Changes in (**a**) CO_2_ assimilation rate (μmol CO_2_ m^−2^s^−1^), (**b**) stomatal conductance (mmol H_2_O m^−2^s^−1^), and transpiration rate (**c**) (mmol H_2_O m^−2^s^−1^) of control and biostimulant-treated cucumber leaves. The asterisk (*) indicates a significant difference (*p* ≤ 0.05) between control and biostimulant and leaves (mean ± s.e.; *n* = 5).

The net photosynthesis (Pn) of cucumber leaves significantly increased on exposure to biostimulant protocol cultivation by 26% compared to the conventional cultivation (Figure 1a). Likewise, the application of those biostimulants caused enhancement of the stomatal conductance and the transpiration rate of the leaves by 35 and 30% of the control, respectively (*p* < 0.05) (Figure 1b,c).

### 2.3. Chlorophyll Fluorescence

**Figure 2:**
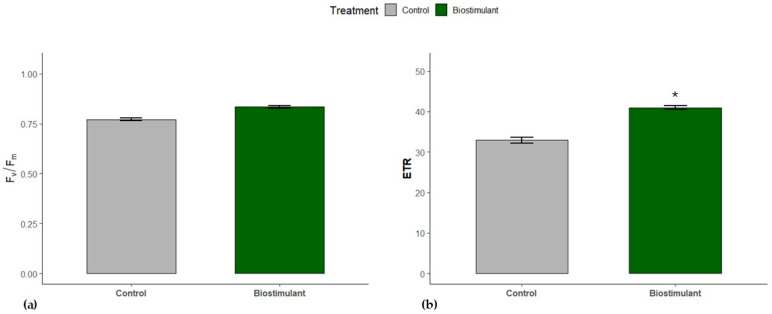
Changes in (**a**) the maximum efficiency of PSII photochemistry (Fv/Fm) and (**b**) the electron transport rate (ETR) of control and biostimulant cucumber leaves. The asterisk (*) indicates a significant difference (*p* ≤ 0.05) between control and biostimulant leaves (mean ± s.e.; *n* = 5).

The electron transport rate (ETR) of the biostimulant-treated leaves showed higher rates compared to the control (*p* < 0.05); the same trend was observed with the maximum efficiency of PSII photochemistry (Fv/Fm) between control and biostimulant leaves (Figure 2a,b).

**Figure 3:**
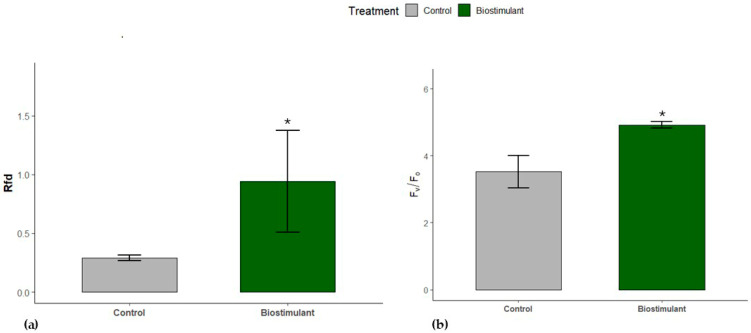
Changes in (**a**) the vitality index (Rfd) and (**b**) the efficiency of the oxygen complex (Fv/Fo) of control and biostimulant cucumber leaves. The asterisk (*) indicates a significant difference (*p* ≤ 0.05) between control and biostimulant leaves (mean ± s.e.; *n* = 5).

Furthermore, after calculating the Rfd (leaf vitality index), leaf biostimulant values were observed to be higher than those of the control group, approximately twice as high, and the difference was statistically significant (*p* < 0.005, Figure 3a). The functionality of the oxygen-evolving complex (OEC, Fv/Fo) of the leaves increased by 38% under biostimulant cultivation compared to the control (*p* < 0.05, Figure 3b).

### 2.4. Fruit Growth, Quality, and Texture

**Figure 4:**
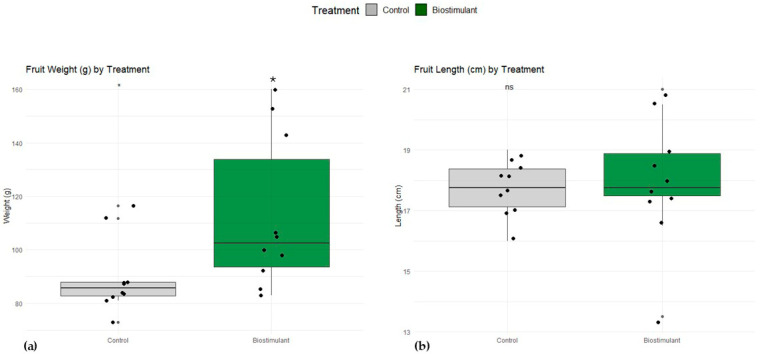
Effect of biostimulant treatment on fruit weight and length. Boxplots show the distribution of (**a**) fruit weight (g) and (**b**) fruit length (cm) for control (gray) and biostimulant-treated (dark green) plants. Individual data points are overlaid as jittered dots. Statistical significance (*p* < 0.05) between treatments was assessed using a *t*-test and is indicated above each comparison (asterisk).

Treatment with the biostimulant significantly increased both fruit weight and length compared to the control. Mean fruit weight for the control group was approximately 91.6 g (range: 72.9–116.5 g), whereas biostimulant-treated fruits averaged 114.3 g (range: 82.8–159.9 g), showing a statistically significant increase (*p* < 0.05). Similarly, mean fruit length increased from 17.8 cm in the control group (range: 16–19 cm) to 18.8 cm in the biostimulant group (range: 13.5–21 cm), also statistically significant (*p* < 0.05). Overall, the biostimulant positively affected both fruit weight and length, indicating improved growth performance relative to the control (Figure 4). Mechanical firmness (flesh resistance) in cucumber fruits harvested at commercial maturity is presented in Table 2. Significant variations were observed among the three points studied in the two treatments. In the control group of fruits, greater resistance was observed near the stem and at their edge (points 1 and 3), while, in the biostimulant fruits, greater resistance was observed in the center (point 2).

More precisely, a non-significant reduction in the flesh resistance near to the stem under biostimulant application by 9% of the control was observed. In contrast, at the middle and the edge of the fruit, significant changes under biostimulants were measured by +12% and −12%, respectively, compared to the controls (*p* < 0.05). In general, the use of biostimulants induced an increase in fruit firmness, indicating denser and mechanically resistant fruits. In contrast, control fruits showed the lowest firmness, suggesting softer textures more susceptible to postharvest damage. A two-way ANOVA showed that the response variable was significantly affected by location (F(2, 24) = 3.98, *p* = 0.032), whereas treatment had no significant effect (F(1, 24) = 0.31, *p* = 0.586). The treatment × location interaction was not significant (F(2, 24) = 0.001, *p* = 0.999), indicating that the effect of treatment was consistent across locations.

Quality attributes, including sugars, organic acids, and vitamin C, were assessed at commercial maturity (Table 3). Specifically, the sugars analyzed were glucose, fructose, and glycerol. Among these, only glucose exhibited significantly higher concentrations in fruits treated with the biostimulant compared to the control group (*p* < 0.05), whereas fructose and glycerol levels did not differ between treatments. Regarding organic acids, malic and lactic acid concentrations were comparable in both groups. Notably, ascorbic acid (vitamin C) content was approximately twofold higher in biostimulant-treated fruits relative to the control (*p* < 0.05).

The color attributes of cucumber fruits at the stage of commercial maturity, expressed in the CIELAB color space, L* (lightness), a* (red-green component), and b* (yellow-blue component), have also been determined (Table 3). L* values of biostimulants were lower, showing a darker color compared to the control, which were lighter, indicating brighter green hues, possibly associated with thinner cuticles or lower chlorophyll density. As cucumbers ripen, they can develop yellow coloration; therefore, a lower positive b* is characteristic of a fresh cucumber, as is observed in biostimulant fruits; however, all the examined color parameters did not differ significantly.

### 2.5. Soil Analysis and Nematode Effects on Roots

**Figure 5:**
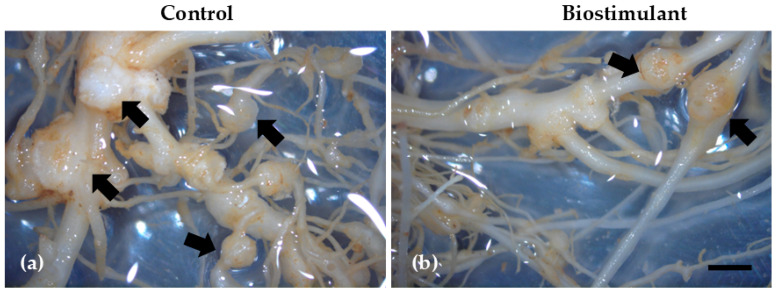
Galls (arrows) in the roots of *Cucumis sativum* L. in control (untreated) (**a**) or biostimulant-treated plants (**b**). In the biostimulant-treated plants, gall number is significantly reduced. Scale Bar: 2 mm.

**Figure 6:**
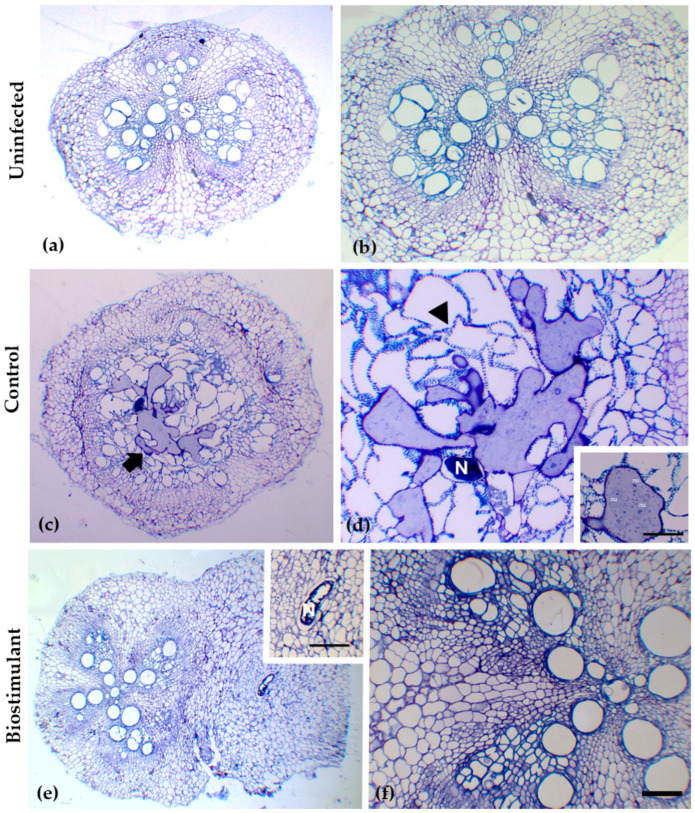
Transverse sections of *Cucumis sativus* L. roots showing anatomical responses to nematode infection under different treatments. (**a**,**b**) Uninfected root segments displaying normal vascular organization and intact cortical tissue. (**c**,**d**) Control (infected) roots exhibiting pronounced structural disruption, including the formation of giant cells or/and syncytia and extensive hypertrophy of cortical and vascular tissues. Arrows indicate giant cells or/and syncytia; arrowheads denote the disrupted vascular tissue; N denotes nematode presence. Inset in d indicates a nematode-induced multinucleated cell (nu; nuclei). (**e**,**f**) Biostimulant-treated roots showed reduced tissue deformation compared to the infected control, with smaller or less developed feeding sites. Inset in e highlight localized giant cell or/and syncytia formation absence around the nematode position (N). Scale bars: (**a**–**f**) 60 μm, inset in (**d**): 20 μm, inset in (**e**): 40 μm.

Soil extraction analyses revealed clear differences between the control and biostimulant-treated plots in the corresponding effects on root morphology (Figure 5 and Figure 6, Table 4) and the density and composition of nematode communities (Table 5), as. Although the total number of nematodes per 100 mL of soil was similar between treatments, substantial changes were observed in the proportion of functional groups. The most striking result was a 66% reduction in plant-parasitic nematodes in the treated soil compared with the control, which aligns with the markedly improved condition of the treated root systems. Control soils contained high populations of infective juveniles, and this abundance strongly correlated with the severe pathological symptoms observed in the roots, including extensive galling (Figure 4, Table 4). Microscopic examinations confirmed the presence of numerous giant cells and large syncytia in the cortical and vascular tissues of control roots, often accompanied by collapsed xylem elements indicative of advanced parasitism (Figure 6).

## 3. Discussion

Recent studies indicate that climate change, particularly extreme weather events such as elevated temperatures, together with biotic stressors, can negatively affect the growth performance of vegetable crops cultivated in greenhouses. Indeed, cucumber productivity is known to be constrained by both biotic and abiotic stress factors, which adversely influence plant physiology and ultimately reduce yield potential [24,25]. Based on this premise, we hypothesized that the application of biostimulants could mitigate some of these negative impacts by enhancing physiological processes and plant resilience.

In our study, cucumbers cultivated under a biostimulant protocol exhibited a significant increase in net photosynthesis, stomatal conductance (gs), and transpiration rate (E) of leaves (Figure 1). This observation indicates a dominant effect of biostimulant treatment on photosynthetic performance. We propose that the specific composition of the biostimulants may strengthen the photosynthetic apparatus and thereby enhance overall agricultural productivity. Supporting this hypothesis, flavonoids—known components of biostimulants—have been reported to positively influence stomatal function and general gas exchange during photosynthesis and respiration [26]. Flavonoids exert antioxidant effects that are critical for maintaining stomatal function under oxidative stress caused by reactive oxygen species (ROS) accumulation in guard cells, thereby prolonging stomatal opening. Moreover, flavonoids can modulate ion channel activity via abscisic acid (ABA), enabling more efficient gas exchange without compromising water balance [26,27].

Thus, flavonoids may enhance photosynthetic activity both directly, by protecting the photosynthetic machinery, and indirectly, by supporting stomatal conductivity. This is consistent with our results, where gs and E indices were higher in the biostimulant-treated group. Similar enhancements in photosynthetic rates under various biostimulant treatments have been reported [5,7,28].

Our findings indicate that plants receiving biostimulant treatments exhibited greater photosynthetic efficiency than control plants. The observed enhancement likely stems from both protection of photosynthetic machinery against environmental stress and direct effects of active biostimulant compounds. Notably, the higher efficiency of the oxygen-evolving complex (OEC, Fv/Fo) in PSII observed under biostimulant treatment (compared to control) can be attributed to the Mn content of the products. Manganese serves as a coenzyme in phenolic compound metabolism, thereby reducing ROS accumulation [29], and is a structural component of the Mn_4_CaO_5_ cluster in OEC, which catalyzes water photolysis and oxygen production during photosynthesis [30]. Higher OEC efficiency under biostimulant treatment was accompanied by slightly elevated—but not statistically significant—maximum PSII photochemical efficiency (Fv/Fm) (Figure 2 and Figure 3) [31].

Additionally, treated plants showed a significant increase in electron transport rate (ETR), indicating enhanced PSII efficiency relative to controls. The 20% increase in ETR under biostimulant application may contribute substantially to biomass accumulation. Iron, another component of the biostimulants, plays a critical role in electron transport as a structural component of chlorophyll and is essential for its biosynthesis [32,33,34].

We also observed an increase in the photosynthetic vitality index (Rfd = (Fm − Fs)/Fs), which integrates the efficiency of primary photochemical events with Calvin cycle activity [10,35]. The significant increase in Rfd under biostimulant treatment reflects improved photosynthetic capacity and CO_2_ fixation rates. Collectively, these findings suggest that biostimulants influence photosynthetic function both at the PSII reaction center, associated with electron transport, and at the OEC water-splitting site, or possibly at both sites simultaneously.

Zinc, present in KeyPlex 120^®^, further supports photosynthetic function by stabilizing chloroplast membranes and participating in chlorophyll metabolism [36]. It also contributes to the biosynthesis of phenolic compounds and lignin in cell walls, enhancing structural resistance to mechanical damage [36]. Humic acids, in combination with micronutrients such as Fe, Mn, and Zn, are likely to improve nutrient availability and utilization, which in turn enhances photosynthetic efficiency, defense mechanisms, and overall plant vitality and productivity [37,38]. Previous studies have similarly reported that biostimulants can influence chlorophyll fluorescence and photosynthetic activity through improved nutrient availability [5,10,39,40].

The biostimulant significantly increased both fruit weight and length, suggesting enhanced growth and development. These results are consistent with reports that biostimulants can improve nutrient uptake and stimulate plant metabolism, leading to greater biomass accumulation and fruit size. Although some variability was observed, the treatment effectively enhanced fruit performance, highlighting its potential for improving yield and quality in cultivation. Beyond photosynthesis, textural characteristics of fruits are determined by cellular structural and biochemical properties, including cell wall strength and elasticity, cellular arrangement, turgor, and cell-to-cell adhesion. In cucumbers, hardness is a primary quality parameter because the fruit is consumed raw [41]. In our study, fruits from biostimulant-treated plants displayed higher flesh resistance at the mid-fruit region, associated with better cellular swelling and nutrient filling. A mesocarp exhibiting a denser texture, with fewer cavities and spaces, likely reduces moisture loss and improves resistance to mechanical stress and pathogens [42]. Flavonoids in the biostimulants may have contributed to the maintenance of cell wall structure and increased vitamin C content (ascorbic acid), a non-enzymatic antioxidant capable of mitigating ROS and preventing cellular damage [26]. Enhanced vitamin C levels in treated fruits correlated with higher leaf CO_2_ assimilation rates and Rfd values. Moreover, biostimulants influenced sugar metabolism, with higher glucose concentrations detected, although organic acid levels remained unchanged. These biochemical changes may improve fruit flavor and overall nutritional quality [43,44].

Skin color is another key indicator of cucumber fruit quality [45,46]. Instrumental color measurements (L*, a*, b* values in the CIELAB system) showed a slight, non-significant reduction in L* (darker green) in biostimulant-treated fruits, with no significant differences in a* and b* values between treatments (Table 3). These results align with prior studies, which show that optimized nutrition and appropriate crop density can maximize cucumber fruit coloration in accordance with varietal characteristics [41,47].

Although the experimental duration did not allow for statistically significant changes in soil physicochemical properties, biostimulant application led to a stabilization and slight improvement of organic matter and microbial indices. This suggests a potential long-term regulatory effect on soil health, which warrants further investigation over extended cropping cycles [48]. Notably, biostimulant-treated plants exhibited a substantial reduction in nematode infestation, indicating that bioactive compounds can modulate nematode life cycles while enhancing plant physiological resilience. Flavonoid-based formulations may have contributed to enhanced rhizosphere signaling and basal immunity, as flavonoids are well-established regulators of plant–nematode interactions and defense priming [20]. These compounds in root exudates can disrupt host-location cues for nematode juveniles, limiting root penetration. Additionally, flavonoids act as modulators of oxidative stress and ROS homeostasis, helping plants counteract nematode-induced cellular damage during early root invasion [26,49]. This biochemical fortification could justify the observed reductions in hypertrophy, diminished formation of giant cells, and preserved vascular architecture in treated roots (Figure 5).

Plant-derived oils in the biostimulants also contributed to protective effects. Their complex mixtures of terpenoids, phenolics, and aromatic compounds exhibit nematicidal or repellent activity, as reported against *Meloidogyne* spp. [50,51]. Reductions in egg masses and underdeveloped feeding sites in treated plants support the hypothesis that essential oils interfere with nematode neuromuscular signaling and cuticular integrity [52]. Concurrently, humic acids and micronutrients (Fe, Mn, Zn) in the biostimulant mixtures likely enhanced microbial activity and nutrient availability in the rhizosphere, facilitating natural suppression of parasitic nematodes [23,37]. These amendments stimulated root growth and metabolism, reinforcing both physical and biochemical barriers to nematode attack. Organic amendments, including plant-derived compounds, can enhance root architecture and plant vigor while releasing bioactive metabolites that directly suppress nematodes by reducing egg hatching and juvenile survival, thereby limiting root penetration and establishment [53]. From a commercial perspective, biostimulants containing vegetable oils, micronutrients, and flavonoids can be integrated into existing fertigation and foliar spray schedules without major infrastructural modifications. While initial costs may exceed conventional inputs, improvements in yield, fruit quality, and reduced nematicide use can yield favorable cost–benefit outcomes [43]. Importantly, these biostimulants are compatible with integrated pest management (IPM) programs, enhancing plant resilience without negatively impacting beneficial organisms, while their natural or low-residue profiles minimize concerns regarding chemical residues and regulatory restrictions in protected cultivation systems [8].

Although the results consistently demonstrate positive effects of the biostimulant-based protocol, certain experimental limitations should be acknowledged. Control and biostimulant treatments were conducted in two separate greenhouses rather than within a fully randomized single-greenhouse design; thus, minor microclimatic differences (e.g., light distribution, temperature and humidity gradients, and airflow) cannot be entirely excluded, despite identical management and automated climate control systems. Moreover, nematode infestation occurred naturally and was not standardized at the beginning of the experiment, meaning that initial population densities may have varied between greenhouses—a common constraint in applied greenhouse research [48]. Nevertheless, the convergence of independent physiological, anatomical, and biochemical indicators—including photosynthetic performance, root integrity, nematode suppression, and fruit quality—supports the robustness of the observed treatment effects. Future studies employing randomized block designs within single greenhouses and multi-site, multi-season validation trials are recommended to further strengthen causal inference and broader applicability [8,54].

Collectively, these multi-component biostimulants exert a combined and protective effect by exerting diverse modes of action—such as stimulating nutrient uptake, enhancing root development, modulating hormonal responses, and improving stress tolerance pathways—that together enhance plant physiological performance and resilience more effectively than individual components alone [54]. They restrict nematode penetration, limit the development of feeding structures, and preserve vascular tissue integrity. Their indirect benefits on plant physiology—including enhanced photosynthetic performance, improved gas-exchange efficiency, and superior nutrient and water uptake [55,56]—reflect the strengthened functional architecture of the root system. These findings support the growing consensus that biostimulants represent an effective, environmentally sustainable alternative to synthetic nematicides, improving plant–environment interactions without suppressing non-target soil biodiversity [54]. Ongoing research will broaden the understanding of how these biostimulants work and identify new sources and formulations that could further improve their efficacy and cost-effectiveness. To achieve this, future research must include large-scale field trials with multi-season experiments across diverse climatic zones (Mediterranean, temperate, tropical) and soil types (sandy, loamy, clay), enabling a comprehensive evaluation of biostimulant performance under realistic agricultural conditions. In addition, experiments comparing different dosages and application schedules (e.g., early vegetative stage vs. flowering stage) will help determine optimal treatment regimes tailored to crop phenology and stress periods. Analyses extending beyond immediate plant responses to include fruit postharvest storage stability, nutrient retention, and consumer sensory panels will introduce new information on quality and marketability. Building on physiological and biochemical perspectives, recent research on plant–nematode interactions has also shown that certain biostimulant treatments, such as acetic acid, can stimulate natural defense mechanisms by modifying root cell wall components and hampering nematode-induced structures, underscoring the value of mechanistic studies alongside field evaluation [57].

## 4. Materials and Methods

### 4.1. Experimental Design

An experiment was conducted at two separate greenhouses of 250 m^2^ each at Marathon region of Attika, Greece, during the period of June to September 2024, till the commercial maturity of the fruits. Greenhouse conditions were automatically controlled as follows: temperature 15 °C (min), 34.5 °C (max), and 27.3 °C (average); average relative humidity 65%; average photosynthetic photon flux density at leaf level (PPFD) 400 ± 50 μmol m^−2^ s^−1^. In early June, uniform 25-day-old plants at the 4-leaf developmental stage were transplanted into the two greenhouses, each containing 625 plants. Six rows of plants were placed with 40 cm between them and 80 cm of space between the rows. The selected spacing dimensions reflect practical commercial constraints and canopy uniformity. After 10 days of transplantation, a soil fertilization of N-P-K (20-20-20) was applied every 15 days. All plants in both greenhouses were managed under integrated pest management practices, with identical fertilization, irrigation, and pest-control regimens across treatments.

To reduce nematode infestation, two different treatments were applied during cultivation of cucumber plants (*Cucumis sativus* L. cv Murat): The first one, in the first greenhouse (so-called conventional control), the insecticide abamectin was used to control nematodes at a dose of 1 mL/lt in combination with an iron chelate (EDDHA) preparation of 5.5% FE SG 1 g/lt.

The second one, in the second greenhouse, CropBioLife^®^ (1 mL/lL) + KeyPlex 120^®^ (1 mL/L) + Key Eco Oil^®^ (2.5 mL/L) biostimulants diluted in tap water were applied. The application was performed through irrigation on the soil at a dosage of 200 mL Key Eco Oil^®^ every 15 days. Sprays were also carried out with 100 mL of CropBioLife^®^, 100 mL of KeyPlex 120^®^, and 250 mL of Key Eco Oil^®^ per 100 L of water, every 7 days.

In the first greenhouse with *Cucumis sativus* L. plants, the control group was established in which the bioactive products were not administered. On the contrary, conventional fertilization products and insecticides were used, with the aim of targeted observation of root nematodes in relation to the experimental group without the interference of entomological pathologies.

The concentrations of the biostimulants used were selected from preliminary experiments, and some information about them is given below:

Key Eco Oil^®^ is a blend of vegetable oils (10% cinammon oil + 5% thyme oil + 5% rosemary oil + 5% sesame oil (*w*/*w*), a product of the biotechnology company Keyplex, licensed for use in organic agriculture. It combines perfectly with plant protection products in the treatment of sucking insects and mites, thanks to its special composition https://www.mygeogreen.com/product/key-eco-oil) (accessed on 1 December 2025) 

KeyPlex 120^®^ is a formulation of micronutrients (Fe: 0.2%, Mn: 0.11%, Zn: 0.10% *w*/*w*), availability most often found deficient in plants. It also contains humic acid, which may enhance soil micronutrients. KP 120 is formulated for foliar application or fertigation to prevent and correct micronutrient deficiencies when used as directed (https://www.keyplex.com/wp-content/uploads/2020/03/KP-120-Label-1.pdf). (https://www.keyplex.com/product/keyplex-120) (accessed on 1 December 2025).

CropBioLife’s power comes from its unique blend of activated flavonoids, while other dominant nutrients are N:1860 ppm, K:1370 ppm, S:482 ppm, and Na:720 ppm. The cycle of carbon removed from the air and deposited in the soil is the building block of regenerative agriculture. The flavonoids in CropBioLife^®^ work by improving the plant’s metabolism. This triggers an energy boost that enables efficiency in two very important plant functions. Photosynthesis and Root Exudation. The beginning of plant health starts with photosynthesis. By enabling efficient photosynthesis, we can observe a more significant carbon dioxide intake, promoting exponential energy production. The additional energy in the form of carbohydrates enables the plant to increase root exudation, which nurtures the microbial biology in the soil, enabling the plant to cultivate the microbes that provide the key nutrients to the plant’s survival (https://www.cropbiolife.com) (accessed on 1 December 2025) [43].

### 4.2. Soil Sampling and Analysis

Soil samples were collected from the rhizosphere of *Cucumis sativus* L. plants grown in control and biostimulant-treated greenhouse plots. Sampling was carried out at a uniform depth of 0–20 cm and at standardized distances from the root zone to ensure comparability among treatments. For each treatment, three independent subsoil specimens (approximately 100 cm^3^ each) were collected per plant and immediately transported to the laboratory, where they were processed within 48 h.

Soil physicochemical properties were determined at the beginning and at the end of the experiment following standard protocols. Soil texture, expressed as percentages of sand, silt, and clay, was determined using the hydrometer method (Gee and Bauder, 1986) [58]. Soil pH was measured in a 1:2.5 soil-to-water suspension using a calibrated pH meter, while electrical conductivity (EC, mS cm^−1^) was determined in a 1:5 soil-to-water extract using a conductivity meter. Organic matter content was assessed by the Walkley–Black wet oxidation method, and calcium carbonate content was measured using the Bernard calcimeter method. Total nitrogen (TN, %) was determined by the Kjeldahl digestion method. The microbial quotient (qMIC) was calculated as the ratio of microbial biomass carbon to organic carbon [59].

All measurements were performed in triplicate, and data are presented as means ± standard error (SE; *n* = 3 subsoil specimens). Differences between treatments were analyzed using Student’s *t*-test, with statistical significance set at *p* < 0.05.

To evaluate the effects of biostimulant treatments on soil nematode community structure, rhizosphere soil samples were collected from *Cucumis sativus* L. plants grown in control and biostimulant-treated greenhouse systems. Sampling was performed at uniform depths and standardized distances from the root zone to ensure comparability among treatments, following recommended rhizosphere sampling procedures (Bongers and Ferris, 1999) [60]. Approximately 100 cm^3^ of rhizosphere soil was collected per plant and processed within 48 h.

Nematodes were extracted from soil samples using aqueous decanting and sieving, followed by Baermann funnel extraction for 24–48 h. The resulting suspensions were examined under a stereomicroscope, and all nematodes were counted and assigned to functional trophic groups, including plant-parasitic, bacterivorous, fungivorous, omnivorous, and predatory nematodes. Plant-parasitic nematodes were further differentiated into root-knot (*Meloidogyne* spp.) and cyst (*Heterodera* spp.) nematode groups based on microscopic examination of key morphological characteristics. Diagnostic features used for provisional identification included the presence, length, and shape of the stylet; head morphology; body length and shape; and tail morphology, following standard parasitological identification criteria. Root-knot nematodes were identified by their characteristic stylet structure and body morphology, whereas cyst nematodes were distinguished by differences in stylet length, tail shape, and overall body form.

Functional classification and ecological indices were calculated [61].

### 4.3. Gas Exchange—Chlorophyll Fluorescence Measurements

Leaf gas exchange measurements were coupled with measurements of chlorophyll fluorescence using an open gas exchange portable system (LI-6400; LI-COR, Inc., Lincoln, NE, USA) with an integrated fluorescence chamber head (LI-6400-40 leaf chamber fluorometer; LI-COR, Inc.). Measurements on the 3rd fully expanded from the top of the cucumber plant, younger leaves were conducted between 9:00 and 12:00 h. Leaf temperature inside the cuvette was maintained between 25 and 27 °C, and the cuvette relative humidity was about 60%. The CO_2_ concentration at the reference infrared gas analyzer (IRGA) was maintained at 400 μmol mol^−1^ by means of a 12 g CO_2_ cylinder and the 6400-01 CO_2_ injector, with the airflow rate through the chamber maintained at 400 μmol s^−1^. Leaf gas exchange measurements were calculated by the LI-6400 operating software Version 5, according to the method of von Caemmerer and Farquhar [55].

In vivo chlorophyll fluorescence was measured on the upper surface of the 3rd fully expanded, from the top, younger leaves after they were left for 20 min to dark adaptation at greenhouse temperature. Different values were selected to determine any structural and functional changes in the photosynthetic apparatus under different treatments: Fv/Fm, the maximum efficiency of photosystem II (PSII) photochemistry; Fv/Fo, efficiency of the oxygen-evolving complex (OEC); Rfd (fluorescence decrease ratio known as vitality index) = (Fm − Fs)/Fs; and the electron transport rate (ETR) [43]. A total of 5 leaves from different plants of the two treatments were measured. The leaves were sampled from several plants, and they are representative of the foliage position. We chose the third leaf for photosynthesis measurements because it is fully expanded and mature but not yet senescent. In this way it has a high chlorophyll content and actively photosynthesizes.

### 4.4. Growth and Texture Analysis of Cucumber Fruit

The fresh weight and length of 10 cucumber fruits from randomly selected plants and different plant positions at harvest time were measured.

Firmness, or flesh resistance, was measured on the whole cucumber fruits. Penetration was performed at 3 points on each sample as follows: one 2 cm from the stem, one in the middle, and one 2 cm from the edge of the fruit. The maximum rupture force (N) developed during the test was determined using the puncture test performed with a Texture Analyzer (TA. HD plus-Stable Micro Systems Ltd., Surrey, UK) using a needle probe diameter of 2 mm, with a test speed of 2 mm s^−1^, and a penetration distance of 10 mm. A total of five samples per treatment were analyzed immediately after harvest [17].

### 4.5. Ascorbic Acid Content

The ascorbic acid content of cucumber fruits was estimated by macerating the sample mechanically with a stabilizing agent (5% metaphosphoric acid) [62]. Data were expressed as mg 100 g^−1^ FW.

### 4.6. Sugars and Organic Acids

Sugars and organic acids were quantified on an Agilent Series 1100 HPLC system, Agilent Technologies, Waldbronn, Germany, equipped with RID and UVD detectors. The column used was Repromer H, 9 µm, 300 mm × 7.8 mm length. The mobile phase was 1 mM H_2_SO_4_^−^. Column temperature, 50 °C; flow rate, 0.7 mL/min; and injection volume, 20 μL. Three fruit replicates were analyzed for the two treatments. All initial fruits were of comparable size, and half of the fruit was taken for homogenization [63].

### 4.7. Fruit Color

The instrumental color of the cucumbers was measured using a Minolta CR-300 colorimeter (Minolta, Konica Minolta GmbH, Langenhagen, Germany) in the CIEL*a*b* system: red share—a*, yellow share—b*, and brightness—L*. A total of twelve samples per treatment were analyzed immediately after harvest [64].

### 4.8. Macroscopically and Microscopically Root Evaluation

Root systems were harvested and examined both macroscopically and microscopically to characterize the severity of nematode infestation. Macroscopic evaluation was conducted using established gall index rating scales to quantify plant response to root-knot nematodes [65]. Microscopic analyses followed a standardized workflow including tissue fixation, dehydration, embedding, sectioning, and staining, allowing detailed assessment of feeding site morphology and structural alterations within the root cortex and vascular tissues [65,66]. Observations were performed using a Stemi 2000-C stereomicroscope (ZEISS Carl Zeiss AG, Munich, Germany) equipped with an integrated ProgRes C3 digital camera (JENOPTIK, Jenoptik AG, Jena, Germany), and section observations were carried out using an Axioplan microscope (ZEISS) equipped with an integrated AxioCam digital camera (Carl Zeiss AG, Munich, Germany). For each plant (*n* = 5), root segments were randomly selected along the entire root system at equal intervals. The total number of root segments analyzed per plant was *n* = 5. This approach ensured unbiased sampling for subsequent nematode extraction and analysis.

To evaluate the effects of biostimulant treatments on soil nematode community structure, rhizosphere soil samples were collected from *Cucumis sativus* L. plants in both control and biostimulant-treated greenhouse systems. Nematode infection occurred naturally under field/greenhouse conditions, and no artificial inoculation was performed. The initial nematode population density was not experimentally controlled but reflected the naturally occurring infestation at the study site. Sampling was conducted at uniform depths and distances from the root zone to ensure comparability across treatments, following recommended rhizosphere sampling procedures [65]. Rhizosphere soil samples (approximately 100 mL per plant) were collected at a uniform depth and processed within 48 h. Nematodes were extracted by aqueous decanting and sieving, followed by Baermann funnel extraction for 24–48 h. The resulting suspensions were examined under a stereomicroscope, and all individuals were counted and assigned to functional groups, including plant-parasitic, bacterivorous, fungivorous, omnivorous, and predatory nematodes. This classification followed the widely accepted ecological framework [67].

### 4.9. Statistical Data Analysis

All data were statistically analyzed using R software (v. 4.3.1; R Core Team, 2023) [68]. The experiment was arranged in a completely randomized factorial design with two treatments (control—conventional and biostimulant). Data are presented as means ± standard error (SE). Sample sizes were *n* = 3 for soil physicochemical and nematode parameters, *n* = 5 for plant parameters, and *n* = 10 for fruit parameters.

Differences among treatments were assessed using one-way analysis of variance (ANOVA). Comparisons between pre- and post-experiment measurements were performed using two-tailed paired *t*-tests, while comparisons between two independent groups were conducted using unpaired two-tailed *t*-tests, as appropriate. Fruit texture data, influenced by both treatment and sampling time, were analyzed using two-way ANOVA. Statistical significance was set at *p* ≤ 0.05, and statistically significant differences are indicated in the tables and figures.

## 5. Conclusions

Biostimulants have gained increasing global adoption due to their biological origin, which confers low toxicity and rapid environmental degradation. These characteristics position biostimulants as sustainable alternatives to chemical fertilizers, which are associated with significant ecological concerns. This study evaluates the efficacy of a biostimulant-based protocol in greenhouse-grown cucumber (*Cucumis sativus* L.) cultivation. Application of biostimulants enhanced leaf physiological functions and improved fruit quality, resulting in superior plant performance compared to conventional practices. Notably, biostimulant treatment positively influenced electron transport rate (ETR), accompanied by increased photosynthetic rate (Pn), transpiration (E), and improved efficiency of the oxygen-evolving complex, as reflected by a higher vitality index. Although total nematode abundance remained similar between treatments, biostimulant application—particularly with CropBioLife^®^, KeyPlex 120^®^, and Key Eco Oil^®^—resulted in substantial reductions in plant-parasitic nematodes, alongside improved root structure and fewer pathological symptoms. The treatments with bioactive substances not only suppressed nematode infestations but also promoted healthier cucumber growth and development, underscoring their potential as sustainable alternatives for crop protection. Indeed, they resulted in a reduced population of parasitic nematodes and fewer root galls. These findings suggest that biostimulants represent strategic tools for optimizing crop productivity under suboptimal growth conditions. Nevertheless, further research in large-scale field trials is required to establish optimal application protocols and adapt biostimulant use to the specific physiological requirements that may apply to other cucumber cultivars and possibly related cucurbits, pending further validation.

## Figures and Tables

**Table 1:** Soil physicochemical characteristics at the beginning and the end of the experiment.

Treatment	Sampling Time	Soil Texture (Sand–Clay–Silt, %)	pH	EC (mS cm^−1^)	Organic Matter (%)	CaCO_3_ (%)	Total N (%)	qMIC
Control	beginning	78–14–8	7.03 ± 0.04	0.991 ± 0.02	1.12 ± 0.05	5.5 ± 0.3	0.30 ± 0.02	0.30 ± 0.03
Control	end	78–14–8	7.01 ± 0.05	0.998 ± 0.03	1.10 ± 0.06	5.4 ± 0.4	0.29 ± 0.02	0.31 ± 0.03
Biostimulant	beginning	71–19–10	7.12 ± 0.05	0.996 ± 0.03	1.23 ± 0.06	6.2 ± 0.4	0.20 ± 0.01	0.40 ± 0.04
Biostimulant	end	71–19–10	7.15 ± 0.04	1.005 ± 0.04	1.26 ± 0.07	6.3 ± 0.3	0.22 ± 0.02	0.42 ± 0.05

Notes: Values represent means ± standard error (SE) of three independent subsoil specimens (*n* = 3) per treatment. No statistically significant differences were detected either between treatments or between sampling times (*p* > 0.05).

**Table 2:** Fruit firmness in Newtons of mature cucumber fruits.

Fruit Firmness (N)			
	Point 1	Point 2	Point 3
Control	1209.46 a	1150.96 b	1182.60 a
Biostimulant	1104.58 a	1285.80 a	1050.96 b

Values are means of five replicates. Different letters in the same column indicate a statistically significant difference (*p* < 0.05).

**Table 3:** The sugars, the organic acids, the vitamin C content, and the color parameters of control and biostimulant-treated cucumber fruits.

	Sugars (% FW)			Organic Acids (% FW)		Color (CIE Lab)			Vit C (mg 100 g^−1^ FW)
	Glucose	Fructose	Glycerol	Mαlic Acid	Lactic Acid	L*	a*	b*	
Control	1.36 b	1.35 a	0.02 a	0.24 a	2.11 a	30.35 a	−3.62 a	+2.87 a	3.12 b
Biostimulant	1.52 a	1.47 a	0.02 a	0.21 a	2.15 a	29.37 a	−3.45 a	+2.85 a	5.97 a

Notes: Values are means of three replicates for sugars, organic acids, and vitamin C and by twelve replicates for color. Different letters in the same column indicate a statistically significant difference between the control and biostimulant treatment (*p* < 0.05).

**Table 4:** Galls count and dry weight analysis.

Parameter	Control	Biostimulant
Total galls counted (*n*)	526	308
Dry weight of root sample (g)	0.26	0.27
Galls per gram dry weight (galls g^−1^)	2023	1140
Percent reduction vs. control (%)	-	43.61%

Notes: Galls were counted under a stereomicroscope. Dry weight was obtained after oven-drying at 60 °C to constant mass. Galls per gram were calculated as total galls divided by dry mass.

**Table 5:** Nematode populations per 100 cm^3^ soil.

Nematode Group	Control (Count)	Biostimulant (Count)	% Difference
Total nematodes (all groups)	1000	1020	~Equal
Plant-parasitic nematodes	300	102	−66%
Non-parasitic nematodes	140	740	+81% in treated
Bacterivores	350	355	~0%
Fungivores	210	273	+23% in treated

Notes: Nematode abundances represent natural populations, since no inoculation was applied. Although total nematode numbers were comparable among samples, the composition of the nematode community differed substantially.

## Data Availability

The data presented in this study are available in this article.
